# Defining nursing workload predictors: A pilot study

**DOI:** 10.1111/jonm.13523

**Published:** 2021-12-12

**Authors:** Dhurata Ivziku, Federica Maria Pia Ferramosca, Lucia Filomeno, Raffaella Gualandi, Maddalena De Maria, Daniela Tartaglini

**Affiliations:** ^1^ Department of Nursing Innovation and Development Campus Bio‐Medico of Rome University Hospital Rome Italy; ^2^ Department of Biomedicine and Prevention University of Rome Tor Vergata Rome Italy; ^3^ Department of Biomedicine and Prevention University Tor Vergata Rome Italy; ^4^ Department of Health Professions Campus Bio‐Medico of Rome University Hospital Rome Italy

**Keywords:** hospital, nursing, staffing, workflow, workload

## Abstract

**Aim:**

To explore predictors of perceived nursing workload in relation to patients, nurses and workflow.

**Background:**

Nursing workload is important to health care organisations. It determines nurses' well‐being and quality of care. Nevertheless, its predictors are barely studied.

**Methods:**

A cross‐sectional prospective design based on the complex adaptive systems theory was used. An online survey asked nurses to describe perceived workload at the end of every shift. Data were gathered from five medical‐surgical wards over three consecutive weeks. We received 205 completed surveys and tested multivariate regression models.

**Results:**

Patient acuity, staffing resources, patient transfers, documentation, patient isolation, unscheduled activities and patient specialties were significant in predicting perceived workload. Nurse‐to‐patient ratio proved not to be a predictor of workload.

**Conclusions:**

This study significantly contributed to literature by identifying some workload predictors. Complexity of patient care, staffing adequacy and some workflow aspects were prominent in determining the shift workload among nurses.

**Implications for nursing management:**

Our findings provide valuable information for top and middle hospital management, as well as for policymakers. Identification of predictors and measurement of workload are essential for optimizing staff resources, workflow processes and work environment. Future research should focus on the appraisal of more determinants.

## 1 BACKGROUND

Nursing work is complex in nature and capturing its variegation is therefore difficult (White et al., 2015). Previous research estimated nursing workload by calculating nurse‐to‐patient ratios, nursing hours per patient day, or volume of nursing tasks based on patient complexity classifications (Griffiths et al., 2020). Other researchers suggested including non‐patient related activities in the workload measurement (Campos et al., 2018; Duffield et al., 2011). Despite extensive research on nursing workload measurement this remains a hot topic in nursing literature.

Nursing workload was defined as ‘all nursing work that must be carried out over a defined period of time’, (Myny et al., 2011) and was recently described as ‘the amount of time and care that a nurse devotes (directly and indirectly) to patients, the workplace, and professional development’ (Alghamdi, 2016). Systems based on quantification of patient care needs, including patient acuity/intensity, complexity of nursing care, casemix of patient diagnosis, and patient turnover, attempted to estimate the demand for nursing resources and related workload (Fagerström & Vainikainen, 2014; Swiger et al., 2016). An increase in nursing care requests, the number of patients cared for, patient demands, and diagnoses can lead to discrepancy between patient needs and the adequacy of nursing resources and heavier workloads (Duffield et al., 2011; Griffiths et al., 2020). Moreover, increased patient numbers and a heavier patient load limit nurse–patient contact, increase care left undone, and intensify time pressure on nurses and concerns about patient outcomes (Yanchus et al., 2017).

Additionally, evidence is emerging that patient turnover in hospitals is increasing (Blay et al., 2017). Increased admissions, discharges, and transfers were reported to intensify nursing workload, create unstable work environments (Yanchus et al., 2017), and were associated with communication gaps, adverse events, and greater length of hospital stay (Blay et al., 2017). Increased patient turnover might also generate an accumulation of patients on a ward from specialties different to those customary in the unit of care. An increased number of patient specialties can lead to more frequent work interruptions, increased information needs from patients and caregivers, reduced work efficiency, poorer patient outcomes (Congdon et al., 2020), and undoubtedly an increase in the perception of nursing workload. Moreover, coordinating several different physician teams might influence workload (Duffield et al., 2011). The effect of patient casemix, understood as previously described, on perceived nursing workload has, however, barely been identified.

Another factor connected to patient care needs and resources is the development of nosocomial infections. It requires prophylactic measures to prevent or contain the spread, including wearing protective equipment, strictly following decontamination protocols, and the creation of dedicated areas for stocking specific supplies (Giuliani et al., 2018). All these measures involve additional nursing activities that increase perceived workload (Duffield et al., 2011). Caring for one or more isolated patients should therefore be considered when estimating nursing workload.

There is copious research on staffing resources, and on nurse and patient outcomes. Evidence reported significant associations between hospital staffing resources, quality of care, and patient outcomes like mortality or failure to rescue (Driscoll et al., 2018). Nursing resources determine the intensity of nursing work necessary for satisfying patient needs (Swiger et al., 2016), and decreased staffing and skill mix was reported to increase workload, tasks left undone, overtime, work pressure, and concerns about quality of care (Duffield et al., 2011; Yanchus et al., 2017).

Besides observing patient acuity, nurse‐to‐patient ratio and staffing resources, researchers also observed the amount of activities performed by nurses during their shift, and workflow, to identify connections with workload. Different time studies documented that nurses spent less than 50% of working time caring for patients, while dedicating the rest of their time to documentation, communication, ward rounds, handover, supply stocking and so forth. (Congdon et al., 2020). Therefore, because the majority of nursing time is employed away from the patient (Congdon et al., 2020) it is important to identify workflow and to evaluate its connection with perceived nursing workload.

Nursing activities were classified as activities connected to patient care (directly or indirectly), unit‐related activities, miscellaneous work and nurses' personal time during a shift (Lavander et al., 2016). Among indirect patient activities, documentation of patient care was reported to occupy a large part of nurses' working time (Duffield et al., 2011). In recent years, the volume of nurse's documentation increased, due in part to increased patient turnover (Blay et al., 2017) or to multiple paper‐based or electronic recording systems (Shihundla et al., 2016). Documentation time might therefore be associated with workload and requires further exploration.

One workflow aspect that might influence nursing workload can be unpredictable events such as patient emergencies or unscheduled patient examinations. These unplanned events are a daily occurrence in nursing work (Fagerström & Vainikainen, 2014). When studying workload, connections between unplanned events and perceived nursing workload should therefore be explored.

With the intention of filling a gap in literature and of revealing prediction effects, this research intends to identify some of the variables associated with nursing workload. Within this study, we test the following hypotheses:

Hypothesis 1 Patient care complexity, number of patients assigned to each nurse (nurse‐to‐patient ratio) and staffing adequacy on shifts are significantly associated with higher workload levels.

Hypothesis 2 Workflow activities related to patient transfers, the number of patients in isolation, presence of patients from different specialties, performing unscheduled activities, information provided to patients or family members, and documentation, all affect the perceived nursing workload.

## 2 METHODS

### 2.1 Design

This research is part of an ongoing multicentre observational study on workload and well‐being. Below we present the pilot study results, which employed a cross‐sectional prospective design.

### 2.2 Theoretical framework

This research was based on the complex adaptive systems (CAS) theory (Holland, 1996). Similarities between CAS and nursing practice have been described (Kiviliene & Blazeviciene, 2019). CAS can therefore be used to understand complex situations, to achieve process optimization, to improve work environments, and to advance nursing science (Kiviliene & Blazeviciene, 2019).

### 2.3 Setting and participants

The pilot study was performed in February 2021 in five medical‐surgical wards of a University Hospital in Italy. We chose random nursing shifts (morning or afternoon) over three consecutive weeks, and at the end of every shift, we asked nurses to complete a questionnaire about the workload perceived. All nurses involved in this study were registered nurses with a university degree in nursing. Some of them also held a master's degree or a clinical specialization. Only full‐time nurses performing direct patient care and working in the ward for at least 2 months were included. Nurses working double shifts or nurses from other services providing support were excluded.

### 2.4 Data collection

All nurses working on the selected shifts and fulfilling the inclusion criteria received a Google Forms link to the survey via their institutional email address. They were identified with a unique numeric code to safeguard anonymity (World Medical Association, 2013), and they could choose whether to answer the entire questionnaire or parts of it.

Nurses were asked to provide demographic details, information about their work experience, their perception of staff adequacy on the shift, the number of patients each nurse was caring for, the number of isolated patients, the number of patients from different specialties, and the patients' care complexity expressed in a rating from 0 (*no complexity*) to 4 (*high complexity*).

Workflow aspects were examined. Nurses were asked to report their involvement in the transfer of patients within and between wards. Unscheduled activities explored were related to unscheduled tests or examinations. Aspects related to providing information to admitted patients and their family members, and to documentation, were also investigated. These aspects of workflow were measured on a 5‐point Likert scale using single items purposely developed for the study, where 0 refers to no nurse involvement in the activity and 4 to high nurse involvement.

To measure perceived workload, we developed a general single item with a 5‐point Likert scale answer option where 0 refers to high workload and 4 to no workload. We chose to measure workload and other workflow predictors with a general single‐item measurement based on literature supporting the use of single item measurements to explore issues in different constructs, and main effects in a reduced number of questions (Diamantopoulos et al., 2012). Research in different fields documented comparable or equal predictive validity when using single‐item scales compared with multiple item measures (Hoeppner et al., 2011).

### 2.5 Ethical considerations

This research received the approval of the local Ethics Committee. The researchers approached the participants individually, explaining the aims of the study, and asked them to sign a written informed consent. Those who refused to sign the informed consent were excluded from the study.

### 2.6 Data analysis

Descriptive statistics, frequency, percentage, means, standard deviations, and chi square tests were performed to describe the participants' characteristics and variables studied. Preliminary data analysis was performed to test assumptions (Alexopoulos, 2010; Byrne, 2013). Using maximum likelihood (ML) estimation and structural equation modelling (SEM), we fitted multivariable linear regression models to identify workload predictors. Variables entered in the model were chosen according to theoretical importance. Three distinct SEMs were tested: one to identify the association between perceived nurse workload, patient acuity, staffing adequacy and nurse‐to‐patient ratio; one to identify the association between workload and patient isolation, specialties, transfers, information, documentation and unscheduled activities; and one to control the final trimmed model using covariates. To evaluate model fit, we used several goodness‐of‐fit indices (Byrne, 2013; Hu & Bentler, 1999). Regression parameters were presented with unstandardized and standardized coefficients. The coefficient of determination (*R* ^2^) was also reported. Statistical tests were two‐sided; *p* values <.05 were considered significant.

It was estimated that a sample size of 125 participants could achieve 95% power to conduct a multivariable linear regression analysis using six predictors with anticipated effect size of 0.10 and a level of significance *p* < .05. However, we enrolled 205 participants for a more stable analysis. Sample size was calculated using G*Power 3.1 (Heinrich Heine University). IBM SPSS Statistics v. 25 and MPLUS v. 8.4 were used to perform analysis.

## 3 RESULTS

Overall, we received 205 completed surveys (response rate 91.5%). Morning and afternoon shifts were equally represented. A substantial number of nurses (37.1%) had up to two years' work experience. The most documented nurse‐to‐patient ratio was from 1:8 to 1:10 (66.4%) and high complexity in patients (55.6%) was reported. Nurses perceived high (59.0%) or medium (37.6%) workload. Other details are presented in Table 1.

**TABLE 1:** Descriptive characteristics of the sample and variables studied (*N* = 205 surveys)

Variables	Mean ± SD (range)	*N* (%)	*p* value
Gender			
Male		10 (4.9)	
Female		195 (95.1)	
Other		0 (0)	
Shifts worked			
Morning shift		106 (51.7)	
Afternoon shift		99 (48.3)	
Work experience in months	79.4 ± 66.1 (2–312)		
0–24 months		76 (37.1)	
25–60 months		25 (12.2)	
61–120 months		45 (21.9)	
>121 months		59 (28.8)	
Nurse‐to‐patient ratio	1:8.6 ± 1.5 (5–15)		
1:5–7 patients		46 (22.4)	.417
1:8–10 patients		136 (66.4)	
1:11–15 patients		23 (11.2)	
Patient acuity	2.7 ± 0.8 (0–4)		
Not at all/a little		8 (3.9)	.147
On average		83 (40.5)	
Enough/a lot		114 (55.6)	
Patient in isolation	0.8 ± 1.0 (0–4)		
0		97(47.3)	.066
1		59 (28.8)	
2		36 (17.6)	
3		11 (5.4)	
4		2 (1.0)	
Patient specialties	2.5 ± 1.1 (0–6)		
≤2		109 (53.2)	.223
3–4		87 (42.4)	
≥5		9 (4.4)	
Patient transfers	1.1 ± 1.2 (0–4)		
Not at all/a little		142 (69.3)	.444
On average		31 (15.1)	
Enough/a lot		32 (15.6)	
Informing patients/family members	1.9 ± 1.0 (0–4)		
Not at all/a little		71 (34.6)	**.030**
On average		78 (38.1)	
Enough/a lot		56 (27.3)	
Health care documentation	2.6 ± 1.0 (0–4)		
Not at all/a little		22 (10.7)	**.001**
On average		64 (31.3)	
Enough/a lot		119 (58.0)	
Unscheduled activities	1.4 ± 1.1 (0–4)		
Not at all/a little		120 (58.5)	**<.001**
On average		53 (25.9)	
Enough/a lot		32 (15.6)	
Adequacy of staff in the shift	1.9 ± 0.9 (0–4)		
Not at all/a little		64 (31.2)	**.003**
On average		96 (46.8)	
Enough/a lot		45 (22.0)	
Perceived nursing workload	1.2 ± 0.8 (0–3)		
Not at all/a little		7 (3.4)	**<.001**
On average		77 (37.6)	
Enough/a lot		121 (59.0)	

*Notes*: *p* value refers to *χ* ^2^ test confronting indicated variables with work experience; in bold significant values.

### 3.1 Assumptions testing

All variables were distributed normally. No missing data were recorded. Assumptions testing for regression analyses showed no multicollinearity and correlations did not exceed the cut‐off point of 0.80 (Vatcheva et al., 2016). Correlations between workload and the determinants explored are presented in Table S1.

### 3.2 Variables associated with nursing workload

Different multivariable models were tested. Because the first model was saturated (0 degrees of freedom) and the nurse‐to‐patient ratio effect was not statistically significant, we specified a new model, removing the insignificant variable. Patient acuity and staffing adequacy were confirmed as variables associated with the perceived workload.

In the second model, which considers workflow variables, patient isolation, specialties, transfers, documentation and unscheduled activities were significantly related to workload. Insignificant association was found between workload and the variable information.

In the third model, we introduced the nurse work experience covariate. All variables, except patient specialty, were confirmed to be significantly associated with nursing workload. The fit indices of the models tested are presented in Table S2. The trimmed models respectively explained 45%, 25% and 26% of the variance in workload. Results of the multivariable regression models are presented in Table 2.

**TABLE 2:** Multivariable regression effects of variables on nursing workload (*N* = 205)

Model 1	*b*	*β*	SE	*p* value
Patient acuity	−0.571	−0.563	0.053	<.001
Adequacy of staffing in the shift	0.186	0.213	0.051	<.001
**Model 2**				
Patient isolation	−0.152	−0.178	0.058	.002
Patient specialties	−0.115	−0.157	0.073	.031
Patient transfers	−0.154	−0.225	0.066	.001
Health care documentation	−0.175	−0.209	0.065	.001
Unscheduled activities	−0.120	−0.158	0.063	.013
**Model 3**				
Nurse working experience	0.004	0.137	0.067	.040
Patient isolation	−0.147	−0.171	0.068	.012
Patient specialties	−0.082	−0.111	0.086	.197
Patient transfers	−0.161	−0.233	0.077	.002
Health care documentation	−0.167	−0.204	0.073	.005
Unscheduled activities	−0.180	−0.242	0.074	.001

*Notes*: Model 1: *R* ^2^ = .448; Model 2: *R* ^2^ = .251; Model 3 with nursing working experience as covariate: *R* ^2^ = .262; *R* ^2^ scores were significant, *p* < .05.

Abbreviations: *R* ^2^, coefficient of determination; *b*, unstandardized coefficient; *β*, standardized coefficient; SE, standard error.

The models tested showed that workload was significantly associated with patient acuity (β = −0.563), adequacy of staffing resources (β = 0.213), patients in isolation (β = −0.171), patient transfers (β = −0.233), documentation (β = −0.204) and unscheduled activities (β = −0.242).

## 4 DISCUSSION

This study explored aspects of patient and workflow to identify variables associated with nursing workload. We identified significant prediction effects of patient complexity and staffing on workload, supporting previous research (Congdon et al., 2020; Qureshi et al., 2020) and improving knowledge on the phenomenon by describing observed effects. Our results indicate that patient acuity and staffing are important aspects to consider when analysing nursing workload and determining staffing requirements. Patient complexity embodies the need for nursing care, and its variation across shifts captures the significance of direct care in workload (Arsenault Knudsen et al., 2018). Additionally, we found that higher staffing is associated with lower nurse workload and identified better prediction effects than those reported in previous literature (Oppel & Mohr, 2021). Insufficient staffing resources were found to predict job dissatisfaction (Hegney et al., 2019) and nurse burnout (Yanchus et al., 2017), and the combination of exiguous staffing and increased workloads were related to poor quality of care (Yanchus et al., 2017). Relational climate (Arsenault Knudsen et al., 2018) and teamwork (Duffield et al., 2011) can mitigate these negative effects. Actions to support teamwork in medical–surgical wards are therefore critical when persistent high workloads are perceived (Yanchus et al., 2017).

Nurse‐to‐patient ratio was not an antecedent of workload in our sample. This finding contributes to existing literature by confirming that perceived nurse workload is not an automatic consequence of nurse‐to‐patient ratio (Oppel & Mohr, 2021). Although nurse‐to‐patient ratio was connected to unfavourable nurse outcomes, job dissatisfaction (Shin et al., 2018) and quality‐of‐care issues, no previous associations with job stress or workload were identified (Oppel & Mohr, 2021). Moreover, our results contribute to the literature dealing with methods for determining staffing requirements (Griffiths et al., 2020), confirming that nurse‐to‐patient ratio is not a sufficiently accurate indicator for decision‐making with regards to staffing. A recent scoping review (van der Mark et al., 2021) reports that perceived adequacy of staffing by nurses could potentially be an available measure for staffing requirements. Our findings support this study. Therefore, as in Oppel and Mohr (2021), perception of staffing resources adequacy is probably a better indicator than nurse‐to‐patient ratio for measuring nurse workload and staffing needs.

Workload was predicted by patient transfers. This finding confirms previous literature and adds information about observed effects. Transferring patients was reported to be time consuming, disruptive to workflow and burdensome for nurses (VanFosson et al., 2017; Yanchus et al., 2017). Considering that at least two nurses are required for a bed transfer, when measuring nurse workload and defining staffing resources, the rate of patient transfers within and between wards should be taken into consideration.

Increased patient turnover will result in considerable nursing documentation (VanFosson et al., 2017). Documentation was associated with workload in previous studies (Moore et al., 2020; Myny et al., 2012) and this is also supported by our findings. Nurses dedicate considerable amounts of time to documentation (Moore et al., 2020) and when a patient's documentation is unavailable, or incomplete, this gives rise to additional nursing time, amplifying an already persistent workload (Shihundla et al., 2016). Nurse workload quantification systems should therefore include documentation.

Patient isolation was confirmed as a workload predictor. When the number of patients in isolation on a ward increases, the perceived nurse workload also rises. This means that when assisting different patients in isolation, nurses are obliged to ration other patients' care; this results in disrupted continuity and quality of care (Hessels et al., 2019). Workload and staffing measurements must, therefore, take our findings into account.

Performing unscheduled activities was another antecedent identified. Previous literature described workflow disruptions and time issues faced by nurses when work routines were fragmented by unexpected events (Fagerström & Vainikainen, 2014), unpredictability of patient casemix or staffing, or when the ward was unstable due to incoming or outgoing transfers (Duffield et al., 2011). Our findings support the literature by identifying significant prediction effects.

Caring for patients of different specialties affects workload. Previous literature reported that an increased length of stay in hospital will increase patient transfers, generating an increased number of specialties within wards (Duffield et al., 2011). Moreover, communication with different physician teams may generate communication gaps, workflow disruption and workload (VanFosson et al., 2017). Our results confirm findings in previous literature and add information about observed effects. In contrast to the other variables, this prediction value disappeared when nurses' work experience was added into the model as a covariate. Literature reported that individual characteristics of nursing staff (like education, skill and experience) improve performance, work engagement (Wang et al., 2021) and that more experienced nurses should report lower workloads (Neill, 2011). We supposed that work experience hinders workload perception in general and that more experienced nurses are better at dealing with the disruptions generated by multiple patient specialties. More studies are therefore needed to explore this phenomenon and to confirm or reject our findings.

Our analysis ruled out the hypothesis that giving information to patients or family members is an antecedent of nurse workload. Qualitative studies described how nurses use snippets of time for communication with patients and families, and how essential these moments were for quality of patient care (Chan et al., 2013). On the other hand, giving information might generate interruptions to nursing work (Myny et al., 2011). This was not the case with our sample. It can be justified by the fact that data were gathered during the COVID‐19 pandemic and family members were not allowed to visit patients. Consequently, nurses might have perceived fewer disruptions to workflow owing to information seeking. Further research to uncover possible predictive effects of this variable is recommended.

### 4.1 Limits and strengths

Although innovative, this study presents some limitations. It is an exploration of pilot data gathered in a single hospital. Even though we included nurses from different wards, our results might be difficult to generalize and should be read with due caution. Additionally, despite our efforts to gather data connected to specific shifts, the observational design of the study means that it is not possible to demonstrate any cause‐effect relationships.

The study presents different strengths, however, in terms of advancing the literature on nursing workload in numerous ways. In contrast with all previous studies, the perceived workload of nurses in this study was connected to specific shifts and therefore more objectively reflected nurses' perceptions. Moreover, we were able to test different variables and identify significant prediction effects on workload contributing to nursing workload research.

Future research is needed to confirm our findings and to explore other workflow aspects such as interruptions, patient admissions and discharges, or nurse involvement in ward management activities. Their effects on perceived workload should then be measured. Additionally, human factor research indicates that workload can affect physical, emotional and psychological aspects of a person. Future research should therefore identify determinants of nursing workload specific to each of these aspects.

## 5 CONCLUSIONS

Nursing workload is an essential part of nursing literature. It helps estimate required staffing resources and is linked to nurse and patient outcomes, and quality of care. Despite its importance, measuring nurse workload is difficult, and the definition of its predictors is still in its infancy. Our research contributes to filling in the literature gap by identifying some patient and workflow predictors of perceived workload. Our findings provide valuable information for top and middle hospital management, as well as for policymakers, regarding the importance of perceived workload for staffing resources.

### IMPLICATIONS FOR NURSING MANAGEMENT

Present national regulations and top management decisions on staffing resources are based on nurse‐to‐patient ratio or nursing hours per patient day indicators. Our findings suggest that managers should calculate the resources needed to guarantee care standards based on indicators of patient complexity and nurse work experience. Moreover, middle management should consider ward workflow aspects when determining staffing assets. Therefore, measuring and analysing workload determinants are essential for developing flexible solutions capable of responding to increased shift workloads on wards.

## AUTHOR CONTRIBUTIONS

All the following authors are entitled to authorship of the article and meet the criteria for authorship, in particular:

- Dhurata Ivziku, PhD, was responsible for the conception and design of the study, acquisition, analysis and interpretation of data; drafted; and critically reviewed the manuscript for important intellectual content;
- Federica M. P. Ferramosca, PhD candidate, contributed design of the study, data analysis and interpretation and critically reviewed the manuscript;
- Lucia Filomeno, RN, contributed to data acquisition and analysis; drafted; and critically reviewed the manuscript;
- Raffaella Gualandi, PhD, contributed to the data analysis and interpretation, and critically reviewed the manuscript;
- Maddalena De Maria, PhD, contributed to the data analysis, interpretation, and critically reviewed the manuscript;
- Daniela Tartaglini, Associate Professor, contributed the data interpretation, and critically reviewed the manuscript for important intellectual content.

All the presenting authors approved the final version of the manuscript and agree to be accountable for all aspects of the work.

The paper has been professionally proofread.

## CONFLICT OF INTEREST

None reported.

## FUNDING INFORMATION

The authors received no financial support for this research.

## ETHICS STATEMENT

The study was approved from the Ethics Committee of University Campus Bio‐Medico of Rome in 9 November 2020 with the protocol number Prot.: 95/20 OSS ComEt CBM.

## Supporting information


**Table S1.** Bivariate correlation coefficients for the included variables.
**Table S2.** Fit indices for the models tested (n = 205 surveys)

## Data Availability

The data that support the findings of this study are available from the corresponding author upon reasonable request.
