# Supplementary material for: Defining nursing workload predictors: A pilot study
Source: J Nurs Manag. 2021 Dec 12;30(2):473–81. doi: 10.1111/jonm.13523 (PMC9300160; doi:10.1111/jonm.13523)
Supplement: Supplementary file 1 — Table S1. Bivariate correlation coefficients for the included variables. Table S2. Fit indices for the models tested (n = 205 surveys) [file JONM-30-473-s001.docx]

SUPPORTING INFORMATION FOR REVIEW AND ONLINE PUBLICATION ONLY

**Manuscript Title: Defining nursing workload predictors: a pilot study preliminary analysis**

**Supporting information 1**

**Table 1.** Bivariate correlation coefficients for the included variables.

|  | 2 | 3 | 4 | 5 | 6 | 7 | 8 | 9 | 10 | 11 |
| --- | --- | --- | --- | --- | --- | --- | --- | --- | --- | --- |
| 1. Nursing workload | -.639** | -.274** | -.266** | -.247** | -.235** | -.247** | -.317** | -.269** | .414** | -.081 |
| 2. Patient acuity | - | .317** | .227** | .146** | .312** | .270** | .338** | .200** | -.356** | .087 |
| 3. Patient Isolation |  | - | .267** | .119 | .017 | -.005 | .136 | .101 | -.312** | .094 |
| 4. Patient Specialties |  |  | - | -.110 | .164* | .194** | .265** | .407** | -.212** | -.055 |
| 5. Patient Transfers |  |  |  | - | .149* | .066 | .033 | -.179* | -.034 | .075 |
| 6. Patient Information |  |  |  |  | - | .167* | .344** | .124 | -.141* | .044 |
| 7. Unscheduled activities |  |  |  |  |  | - | .215** | .202** | -.271** | .000 |
| 8. Healthcare Documentation |  |  |  |  |  |  | - | .238** | -.218** | -.097 |
| 9. Nurse-to-patient ratio |  |  |  |  |  |  |  | - | -.497** | -.088 |
| 10. Adequacy of staffing in the shift |  |  |  |  |  |  |  |  | - | -.062 |
| 11. Nurse Working Experience |  |  |  |  |  |  |  |  |  | - |

**Correlation is significant at the 0.01 level (2-tailed); *Correlation is significant at the 0.05 level (2-tailed).

**Supporting information 2**

**Table 2.** Fit indices for the models tested (n=205 surveys)

| MODELS TESTED | df | χ2 values | | CFI | TLI | RMSEA values | | | SRMR |
| --- | --- | --- | --- | --- | --- | --- | --- | --- | --- |
|  |  | χ2 | p(χ2) |  |  | RMSEA | 90% CI | P value |  |
| 1 | 1 | 1.330 | 0.249 | 0.997 | 0.991 | 0.040 | 0.000-0.195 | 0.362 | 0.022 |
| 2 | 1 | 1.768 | 0.184 | 0.985 | 0.912 | 0.061 | 0.000-0.208 | 0.290 | 0.026 |
| 3 | 5 | 3.633 | 0.603 | 1.000 | 1.000 | 0.000 | 0.000-0.096 | 0.767 | 0.021 |

*Abbreviations*: df: Degree of Freedom; χ^2^: chi-square test; CFI: Comparative Fit Index; TLI: Tucker-Lewis index; RMSEA: Root Mean Square Error of Approximation; CI: 90% Confidence Interval; SRMR: Standardized Root Mean Square Residual.
